# What Would Make Getting Colorectal Cancer Screening Easier? Perspectives from Screeners and Nonscreeners

**DOI:** 10.1155/2012/895807

**Published:** 2012-01-04

**Authors:** Gilda G. Medina, Amy McQueen, Anthony J. Greisinger, L. Kay Bartholomew, Sally W. Vernon

**Affiliations:** ^1^School of Public Health, The University of Texas-Houston, 1200 Herman Pressler, Houston, TX 77030, USA; ^2^Washington University School of Medicine, 4444 Forest Park Avenue, St. Louis, MO 63110, USA; ^3^Kelsey Research Foundation, 5615 Kirby, Houston, TX 77005, USA

## Abstract

*Background*. Despite the availability of multiple effective tests for colorectal cancer (CRC), screening rates are low. Greater understanding of barriers between screeners and nonscreeners may improve public health initiatives to increase CRC screening (CRCS). *Methods*. We conducted a content analysis of 625 responses to the question: “*Was there anything that would have made getting tested easier?*” Respondents were patients at a multispecialty practice who participated in a behavioral intervention trial to increase CRCS. Using clinic records, we classified patients as early-screeners (<6 months), late-screeners(6–12 months), and nonscreeners (>12 months). *Results*. Both screeners and nonscreeners reported the same categories of barriers. However, early-screeners predominantly cited dislike of test attributes such as bowel preparation, whereas nonscreeners cited concerns regarding finances and work and family responsibilities. *Conclusion*. Multilevel strategies that address scheduling barriers and external screening barriers may improve CRCS. Future studies may test hypotheses about mediators explaining how screeners overcome barriers.

## 1. Introduction

Colorectal cancer (CRC) is the third most common cancer in men and women in the USA [1]. Regular CRC screening (CRCS) is recommended beginning at age 50 for average-risk adults [1, 2]. Despite the availability of multiple tests for the early detection and prevention of CRC, screening rates are less than optimal [3, 4].

Considerable research including data from national surveys [5–10] and local studies [11, 12] have described reasons for not undergoing CRCS, but all were based on interviews with nonscreeners. National survey data from 2000 to 2005 consistently found that the two main reasons nonscreeners gave for not having CRCS were lack of awareness of the need for the test and lack of provider recommendation or order [5–10]. Other barriers identified in local studies of nonscreeners include absence of symptoms, being too busy, other health concerns, and logistical problems [11, 12]. Lack of awareness was not a primary reason given for not being screened, perhaps because most of the samples were patients within healthcare settings.

Very few studies have compared reasons given by screeners and nonscreeners in the same study sample [13–15], and findings were somewhat inconsistent. Greater understanding of the similarities and differences in the experience and concerns of screeners and nonscreeners may have important implications for interventions, patient-physician communication, and healthcare system practices and policies designed to increase screening. The purpose of this paper is to extend past research by investigating barriers to screening among patients from a single healthcare system who participated in a behavioral intervention trial to increase CRCS and who subsequently either did or did not complete screening.

## 2. Methods

### 2.1. Setting

The trial was conducted at Kelsey-Seybold Clinic, a large, multispecialty medical group practice in Houston, Texas, by the Kelsey Research Foundation and the University of Texas School of Public Health (UTSPH) (5R01CA097263; PI: Sally W. Vernon). The institutional review board at the UTSPH approved the trial.

### 2.2. Patients and Procedures

A data programmer at the foundation searched the clinic's administrative database to identify patients eligible for the trial with the following characteristics: received primary care at the clinic within the last year, were between 50 and 70 years of age, never had CRC or polyps, had never been screened or were due for CRCS according to American Cancer Society guidelines in effect at the time of the study [16], and had not had a physical exam within the past year. Other eligibility criteria were that patients had no prior diagnosis of Crohn's disease or ulcerative colitis; were English-speaking; had a wellness exam scheduled or were willing to schedule one; were willing to complete a baseline survey; agreed to come to the clinic 45 min before their exam to complete a study visit.

University research staff administered the baseline telephone survey and randomized patients to one of three study groups. All patients met with research staff approximately one hour prior to a physician visit for a wellness exam to review intervention materials, if applicable. Data was collected in 2004–2007. Patients were surveyed by telephone at baseline and 6 months. Additional details about the trial are reported elsewhere [17].

### 2.3. Measures

All participants were asked the following open-ended question on the 6-month survey: “Was there anything that would have made getting tested easier?” Research assistants recorded patients' responses using a web-based survey instrument. All responses were brief, consisting of a few words, a phrase, or a short sentence. Patients were not asked to explain or elaborate on their responses.

Screening status was ascertained from clinic records at 6 and 12 months after-intervention [17]. CRCS status was defined as completing one of the following tests post intervention: fecal occult blood test, sigmoidoscopy, colonoscopy, or double-contrast barium enema. Patients were classified as early-screeners (screened by 6 months), late-screeners (screened >6 months but ≤12 months), and nonscreeners (not screened by 12 months). Thus, early-screeners responded to the question after being screened, whereas late-screeners and nonscreeners had not been screened when they answered the question.

### 2.4. Data Analysis

Using ATLAS.ti, one of the authors (GM) conducted a content analysis beginning with an open-coding, iterative review of patient responses to identify an initial code list that classified, summarized and separated patient responses into similar concepts or units [18]. Following a constant comparison method, two of the authors (GM and AM) refined the code list and organized the codes into mutually exclusive categories and subcategories. We then compared the rank order of codes for each category and subcategory by screening status at followup to examine potential differences and similarities in the pattern and frequency of responses for the 3 groups. We chose not to examine codes by intervention group because the trial results showed no differences in screening rates by group [17], and a qualitative study involving a subset of trial participants found no meaningful differences in physician-patient discussions about CRCS by group [19].

## 3. Results

Baseline surveys were completed by 1224 patients, and 1026 of them completed the followup survey at 6 months post intervention. Of the 1026 respondents, we separated responses concerning barriers (*n* = 625), those simply saying “no,” “none,” or “nothing” (*n* = 320), and those respondents with no comments at all (*n* = 81). The three response groups were similar in gender, age, marital status, and employment status (Table 1). African-Americans were more likely than Whites or Hispanics to report no barriers. Those who responded “none” or “nothing” had less education and lower incomes compared with the other two groups. Early-screeners were more likely than late- or nonscreeners to report that nothing would have made screening easier. In contrast, nonscreeners were more likely than early- or late-screeners to report barriers. 

For our main content analysis, we focused on the 625 respondents who reported barriers to CRCS. Our analysis sample was similar to our overall study sample [17] and was predominantly female, 50 to 59 years old, married, employed, had less than a college degree, and had an annual income of $30,000 or more.

We identified two mutually exclusive categories of responses to the question about what would have made getting tested easier: scheduling barriers and screening barriers. Scheduling barriers were sub-categorized into patient-related and system-related barriers. Patients' reasons for not scheduling CRCS were classified as patient-related, whereas responses related to patients' interaction with the clinic were classified as system-related scheduling barriers. Screening barriers were sub-categorized into external and internal barriers. Circumstances occurring or existing extraneous to patients yet exerting a strong influence on their CRCS decisions or actions were classified as external barriers. Responses that referred to patients' commitment to CRCS or their emotional and psychological reactions to CRCS were classified as internal barriers. Overall, early-screeners were least likely and late-screeners were most likely to report scheduling barriers (Table 2). In contrast, early-screeners were most likely and late-screeners were least likely to report screening barriers. Nonscreeners were equally likely to report both types of barriers. 

### 3.1. Scheduling Barriers

#### 3.1.1. Patient-Related Scheduling Barriers

Nonscreeners reported more “work and family responsibilities” and “being too busy” while more early- and late-screeners reported having a pending appointment (Table 2). The most common family responsibility involved providing care for ill spouses, children, and aging parents, some of whom suffered from illnesses such as cancer or Alzheimer's disease. Quotes illustrating patient-related scheduling barriers are included in Table 3.

#### 3.1.2. System-Related Scheduling Barriers

Having difficulty scheduling an appointment was the most frequently mentioned system-related scheduling barrier among early- and late-screeners (Table 2). Waiting or expecting to be called by the clinic was the most frequently reported barrier mentioned by nonscreeners. Specific responses included reports about having trouble identifying a clinic appointment that fit their schedule (8 of 17 early- and late-screeners and 11 of 34 nonscreeners) and concerns about the wait time for getting an appointment (4 of 17 early- and late-screeners and 5 of 34 nonscreeners). One of 7 late-screeners and 5 of 34 nonscreeners reported that the clinic canceled their appointment, but they did not mention whether they or the clinic attempted to reschedule. The remaining 17 participants (4 of 17 early- and late-screeners and 13 of 34 nonscreeners) wanted the clinic to be more active regarding scheduling of appointments. Of 43 participants waiting or expecting to be called by the clinic, 1 of 3 screeners and 6 of 40 nonscreeners wanted the clinic to schedule the visit with the Gastroenterology Department instead of having to schedule it themselves. One late-screener and 7 nonscreeners wanted direct access to colonoscopy after completing a wellness exam rather than having to schedule a consultation with a gastroenterologist prior to scheduling an endoscopic procedure, the protocol in effect during the study. Sample quotes of system-related scheduling barriers are included in Table 3.

### 3.2. Screening Barriers

#### 3.2.1. External Screening Barriers

For nonscreeners, the most frequently reported external barrier was financial or lack of insurance coverage for CRCS; this barrier was the second most frequently mentioned barrier among both early- and late-screeners (Table 2). Although all participants had health insurance at baseline, screeners and nonscreeners still considered CRCS to be too expensive and reported out-of-pocket costs (e.g., copays or deductibles) ranging from a few hundred to a few thousand dollars. Five of the 14 early and late-screeners had a colonoscopy and reported that the copay was unexpectedly high. Two screeners had previously postponed or rescheduled their appointments due to the copay amount. The remaining 7 screeners who thought the cost was too high completed a stool blood test, which is usually covered by insurance, so it may be that they thought the other tests were too expensive.

Medical conditions were the second most often reported external barrier among nonscreeners (Table 2). Of the 56 nonscreeners, 8 reported having heart problems, 10 reported having surgeries, and 15 reported chronic conditions such as diabetes, arthritis, multiple sclerosis, and cancer. Of the 23 remaining nonscreeners, all reported having acute health concerns that required their immediate attention or financial resources. In contrast, only 4 of the 63 early-screeners who reported external barriers cited medical conditions as a barrier. Although late-screeners ranked medical conditions first among all external screening barriers, only 7 late-screeners reported this barrier.

Dislike of screening test attributes was the most frequently reported external barrier among early-screeners; it was less often mentioned by late- and nonscreeners (Table 2). Overall, 47 of the 65 participants who disliked the screening test attributes specifically disliked the bowel preparation including the large volume of liquid, taste, and laxative effect. Of these 47 responses, 38 were from screeners, which may reflect experience with the test. The remaining 18 participants (7 early-screeners and 11 nonscreeners) reported disliking other CRCS test attributes, namely the invasiveness of endoscopies, the inconvenience of a stool blood test, and in a few cases, sedation. Both early-screeners and nonscreeners reported wanting alternative tests such as a virtual colonoscopy or ultrasound. The least frequently mentioned external screening barriers by all 3 groups were lack of physician direction and transportation needs (Table 2). Only nonscreeners mentioned lack of physician direction as a barrier. Sample quotes illustrating all external personal barriers are included in Table 3.

#### 3.2.2. Internal Screening Barriers

For nonscreeners, the two most frequently reported internal screening barriers were low salience and low perceived need to be screened (Table 2). Participants responded that they had forgotten, remained undecided or had “just not gotten around to it.” In contrast, 3 of 4 early-screeners indicated high salience for CRCS by describing it as a task that simply had to be completed or as one early-screener said, “I did not even stop to think about it—I just got it done.” For early-screeners, the two most frequently reported responses were wanting more information about screening and having emotional concerns such as fear of pain, anxiety about screening, unpleasantness of the test, inconvenience, humiliation, and reluctance to get tested (Table 2). These concerns were less frequently reported by nonscreeners; however, all 3 people who anticipated pain from the test procedure were nonscreeners. All 6 screeners who listed emotional concerns noted that their fears were unfounded due to sedation before the colonoscopy. Quotes illustrating internal barriers are included in Table 3.

## 4. Discussion

Although there was overlap in the rank order of responses from screeners and nonscreeners, we observed some noteworthy differences. For early-screeners, who answered our question after being screened, dislike of test attributes such as bowel preparation, an external screening barrier, was the predominant response. Of 110 early-screeners, 44 said a different preparation or an alternative test that did not involve bowel preparation would have made screening easier compared with 20 of 476 nonscreeners. It is unknown whether bowel preparation was perceived to be a barrier prior to screening by early-screeners in this study, but our findings are similar to other reports in the literature. Although study methods differed, Jones et al. [13] found that bowel preparation ranked first as a barrier for patients who were up-to-date with colonoscopy and for those overdue for sigmoidoscopy or colonoscopy, but not for those never screened. In contrast, Harewood et al. [15] found that not wanting to do the bowel preparation for colonoscopy was the highest ranked reason among both never screeners and among those who had been screened previously. In a study of complications following colonoscopy, 77% of patients reported the bowel preparation as the worst part [20]. In previous studies of nonscreeners only, test preparation was rarely listed among the top five barriers, [11, 12] even in studies that focused on colonoscopy [12]. Although these inconsistent findings may be due to differences in study methods, they leave unresolved the question whether apprehension of or experience with bowel preparation affects both first-time and repeat testing. Providing patients with more detailed information about test attributes and alternatives could help them cope and could increase self-efficacy. Consistent with this idea, screeners reported wanting more information about the screening process.

For nonscreeners, the most frequently cited response was financial and insurance concerns. Very few early-screeners raised this issue (12 of 110 early-screeners). These findings are generally consistent with Jones et al. [13] who found that cost ranked second among screeners and third among those who were overdue. However, Denberg et al. [12] noted that most of the patients who voiced finances as a barrier actually did not know whether colonoscopy was covered by their health insurance plan and the copay amount. Further research is needed to assess patient's willingness to pay for preventive screening.

Nonscreeners frequently mentioned that being too busy and work and family responsibilities were reasons they deferred scheduling CRCS. In contrast, these reasons were infrequently mentioned by early- and late-screeners. Although CRCS does require a time commitment, Denberg et al. [12] speculated that responses from patients about being “too busy” may have obscured motivational barriers. Nonscreeners may benefit from screeners' accounts about how they successfully scheduled and prepared for a test, especially those with more negative feelings about the test procedure or preparation [15].

Compared with screeners, nonscreeners cited medical conditions as an important barrier with many reporting chronic health conditions. Although patients with medical conditions may have more frequent contact with the healthcare system and subsequently more opportunities to be offered screening, prevention may not get addressed. Future research should examine how patients, along with their physicians, prioritize and address multiple health concerns, including preventive health behaviors.

The followup survey may have served as a reminder to nonscreeners to get CRCS [21]. In our study, 39 of 515 nonscreeners at the 6-month survey were later classified as late-screeners because they were adherent by 12-month followup. Late-screeners in our sample may reflect more motivated screeners who were temporarily delayed by barriers. Specifically, patient-related and system-related scheduling barriers were the barriers most frequently mentioned by this group. Other studies also have found scheduling challenges at the system-level [12]. Scheduling issues may reflect patients' lack of flexibility in scheduling appointments, lack of understanding of the screening process, or inability to navigate the healthcare system. Attempts to make CRCS easier for patients such as offering the stool blood test, enabling direct-access colonoscopy, using patient navigators to assist patients through the process, and combining CRCS with other preventive care services (e.g., flu shots [22]) might increase screening rates. Additionally, we found that a small number of patients expected to receive a call from the endoscopy clinic suggesting a desire for more clinic outreach efforts to schedule patients.

In contrast to national data, lack of awareness was not frequently mentioned as a reason not to get screened by any of the 3 groups. Other data from our study showed that physicians consistently brought up CRCS during the exam; however, in general, they did not engage in much discussion or facilitate appointment scheduling [19]. Nevertheless, most patients probably received a recommendation to be screened and were aware of the need to do it. This circumstance may explain our finding that lack of physician recommendation was not cited as a barrier to getting tested, nor did anyone specifically indicate that they were confused by the test options in any of the 3 groups.

CRCS rates are lower among males, whites, and people with lower education, income, and no insurance [23]. Barriers and test preferences may be different among these groups and could be compared in future studies. In our sample, everyone had insurance and baseline preferences by test type and screening rates at 12-month followup did not differ by gender, race/ethnicity, and education [17, 24].

Although we observed interesting differences in the most frequently reported barriers among screeners and nonscreeners, all three groups reported similar categories of barriers. Our results suggest that screeners were better able to overcome some barriers that hindered nonscreeners. Additionally, more early-screeners reported “no” barriers, whereas more nonscreeners reported barriers. Future studies should test hypotheses about mediators or moderators that explain how screeners overcome CRCS barriers. Several limitations should be considered in interpreting our findings. Our question referred to “any” CRCS test. The rank order of responses may have been differed had we asked test-specific questions like Jones et al. [13]. However, our findings suggest that most respondents focused on colonoscopy when discussing barriers to CRCS (especially the scheduling barriers category which is not relevant for stool blood tests). Respondents' focus on colonoscopy is consistent with physicians' consistent recommendations for COL [19] and the fact that most screeners got colonoscopy (57%) [17]. Because most previous studies have only queried nonscreeners, we think it is a strength that our study examined barriers among both screeners and nonscreeners. Further, our open-ended question may have elicited more salient barriers to CRCS irrespective of test type compared with an approach where patients rate a list of investigator-selected barriers. Although the wording of our question allowed us to query both screeners and nonscreeners, the word “easy” may have directed participants' attention to difficulties (e.g., bowel preparation) or barriers (e.g., financial/insurance), rather than to motivational factors or perceived importance of CRCS. Further, early-screeners' responses may be less comparable to nonscreeners because our question did not require them to focus on their barriers prior to screening only. Future studies could examine differences in perceived barriers before and after CRCS to better understand how the procedure influences perceptions. Additionally, we did not probe responses to the open-ended question which may have led to an understanding of how and why screeners overcame their barriers. We also may not have identified all barriers relevant to patients in the lowest category of education and income who were less able or willing to answer the open-ended question. Finally, although many of our findings are consistent with those of other local studies, the generalizability of these findings may be limited because the sample was drawn from a single healthcare system. In terms of our analysis, there was the possibility for misclassification of responses to the open-ended question. However, our team-based approach to coding responses and our process of iterative data analysis, we believe, minimized misclassification.

## 5. Conclusions

Screeners and nonscreeners expressed a range of challenges that may require different solutions including multilevel strategies that address both patient- and system-related scheduling barriers and strategies that address external screening barriers such as cost, dislike of bowel preparation, and medical conditions. Changes at the system-level that may increase CRCS rates include improving scheduling procedures, increasing direct access to colonoscopy, and use of patient navigators. Overcoming external screening barriers like cost will require policy changes that cover copayments while addressing screening barriers such as dislike of bowel preparation may require educational and motivational approaches. Healthcare providers should consider patients' individual needs and barriers when recommending CRCS. In summary, to accomplish the goal of increasing CRCS, it may be necessary to use multiple strategies targeting patients, physicians, and healthcare systems simultaneously.

## Figures and Tables

**Table 1:** Socio-demographic characteristics of survey respondents (*N* = 1026) reporting barriers (*N* = 625), “no” or “nothing” (*N* = 320), or no comment (*N* = 81) on the 6-month followup survey.

	Reported barriers (*n* = 625)		No, nothing (*n* = 320)		No comment (*n* = 81)	
	*n*	%	*n*	%	*n*	%
Gender						
Females	367	58.7	184	57.5	45	55.6
Males	258	41.3	136	42.5	36	44.4
Race/ethnicity						
African-American	273	43.7	164	51.3	35	43.2
Hispanic	74	11.8	22	6.9	7	8.6
White	242	38.7	119	37.2	33	40.7
Other, unreported	36	5.8	15	4.7	6	7.4
Age group						
50–59	497	79.8	261	81.6	63	77.8
60+	128	20.5	59	18.4	18	22.2
Married						
Yes	382	61.1	200	62.5	54	66.7
No	239	38.2	119	37.2	27	33.3
Unreported	4	0.6	1	0.3	0	—
Employed						
Yes	508	81.3	253	79.1	64	79.0
No	114	18.2	64	20.0	16	19.8
Unreported	3	0.5	3	0.9	1	1.2
Education						
Less than college	346	55.4	187	58.4	52	64.2
College degree or higher	276	44.2	130	40.6	29	35.8
Unreported	3	0.5	3	0.9	0	—
Income						
<$30 K	87	13.9	50	15.6	22	27.2
$30 K–<$70 K	284	45.4	137	42.8	27	33.3
$70 K or higher	221	35.4	119	37.2	32	39.5
Unreported	33	5.3	14	4.4	0	—
Screening status						
Early-screener (<6 mo)	110	17.6	180	56.3	34	42.0
Late-screener (>6 mo but ≤12 mo)	39	6.2	12	3.8	0	—
Nonscreener by 12 mo	476	76.2	128	40.0	47	58.0

**Table 2:** Types of scheduling and screening barriers reported by early-, late-, and nonscreeners (*n* = 625).

	Early-screener ≤6 months (*n* = 110)		Late-screener >6 months but ≤12 months (*n* = 39)		Nonscreener by 12 months (*n* = 476)	
	*n*	%	*n*	%	*n*	%
*Scheduling barriers*	27	24.5	30	76.9	233	48.9
	Rank	(*n*)	Rank	(*n*)	Rank	(*n*)
Patient related						
Being too busy	2–3	(3)	4	(3)	2	(50)
Work and family responsibilities	2–3	(3)	3	(5)	1	(61)
Missed appointment	4	(2)	2	(6)	3	(24)
Pending appointment	1	(7)	1	(7)	4	(17)
System related						
Patient had difficulty scheduling appointment with clinic	1	(10)	1	(7)	2	(34)
Patient expected to be called by clinic	2	(2)	2-3	(1)	1	(40)
Patient preferred direct access to colonoscopy	—	(0)	2-3	(1)	3	(7)

	*n*	%	*n*	%	*n*	%
*Screening barriers*	83	75.5	9	23.1	243	51.1
	Rank	(*n*)	Rank	(*n*)	Rank	(*n*)
External						
Financial/insurance concerns	2	(12)	2	(2)	1	(68)
Medical conditions	3	(4)	1	(3)	2	(56)
Patient disliked screening test attributes	1	(44)	3-4	(1)	3	(20)
Perceived lack of physician direction	—	(0)	—	(0)	4	(15)
Transportation needs	4	(3)	3-4	(1)	5	(14)
Internal						
Salience for screening	3	(4)	1	(2)	1	(30)
Perceived low need to screen	4	(1)	—	(0)	2	(23)
Wanted more information	1	(9)	—	(0)	4	(2)
Emotional concerns including fear of pain or discomfort	2	(6)	—	(0)	3	(15)

Barriers are rank ordered within subcategories.

**Table 3:** Selected illustrative quotes.

*Patient-related scheduling barriers*
My own scheduling issues (early-screener).
If I had more flexible times available (late-screener).
I am a teacher. Once the school year has ended, I can set up a time (nonscreener).
My daughter has been ill for the last six months. I have to drive my daughter and I have care-giving duties for my mother (nonscreener).
I have tried to be tested several times but have had to reschedule. I am scheduled for my GI consultation February 13 with Dr. [name withheld] (nonscreener).

*System-related scheduling barriers*
I tried to schedule but a convenient time has not been found (nonscreener).
When I called to get a colonoscopy, they told me to get a consult. I did not see the use of the consult. It could be done right before the colonoscopy (nonscreener).
I'm waiting for them to call me back so that I can get a colonoscopy (nonscreener).

*External screening barriers: financial/insurance barriers*
I would have liked to have known how much it was going to cost. I would not have done it at that time (early-screener).
I had a problem coming up with the deductible, so I had to reschedule (late-screener).
Although I was scheduled, I could not afford the copay (nonscreener).
I changed insurance and the new insurance would not cover as much of it. It would have been very expensive (nonscreener).

*External screening barriers: medical conditions*
Because I had the flu and the doctor directed me to postpone having the colonoscopy (early-screener).
I had a physical problem-bad hemorrhoids (late-screener).
I had a heart condition that required hospitalization and caused me to postpone my plans for screening (nonscreener).
I have had several health problems. I cannot think of colon cancer screening (nonscreener).

*External screening barriers: patients disliked screening test requirements*
Drinking the water/prep was the problem (early-screener).
The laxative stuff is hard to get through (early-screener).
The drink that you have to drink was horrible. If that tasted better (early-screener).
If they had an easier testing process like swallowing a camera. I do not like the purging process (nonscreener).
If I did not have to do the FOBT at all—it was kind of a hassle since I had to do it over 3 days at home (nonscreener).

*External screening barriers: perceived lack of physician direction*
At the physical, the physician did not push me to do any screening (nonscreener).
My doctor's decision would have made it easier, but the doctor is still telling me to wait and see (nonscreener).

*External screening barriers: transportation needs*
I felt that I could have driven myself home after the test. That is what kept me from doing it sooner. I had to find someone to drive me there (early-screener).
I do not have anyone to drive me to the testing for a colonoscopy (nonscreener).

*Internal screening barriers: low salience for screening*
No. It's just me. I'm trying to plan around work and I'm just lazy (late-screener).
Just not on my radar right now (nonscreener).
I have a FOBT kit, but I have not done the test (nonscreener).

*Internal screening barriers: low perceived need for CRCS*
If I thought I had symptoms and was ill then I would have been tested (early-screener).
I do not feel a need for it. My stools are ok and I feel ok (nonscreener).
At the doctor's office, they did a rectal exam. They did not recommend anything further. I thought that was all that was needed (nonscreener).

*Internal screening barriers: information needs*
Maybe seeing a video clip from an actual screening procedure to be better prepared for what is going to happen during the test (early-screener).
I would have liked more information about the procedure (nonscreener).

*Internal screening barriers: emotional concerns*
I did not feel anything during the procedure and I think it would have been easier to get tested if I had known that I really would not have any pain (early-screener).
It would help me complete testing if I could take away some of the fear of the procedure (nonscreener).

GI: gastroenterology; CRCS: colorectal cancer screening; FOBT: fecal occult blood test.
